# Age‐dependent sexual dimorphism in hippocampal cornu ammonis‐1 perineuronal net expression in rats

**DOI:** 10.1002/brb3.1265

**Published:** 2019-03-25

**Authors:** Brian B. Griffiths, Amanda M. K. Madden, Kimbra A. Edwards, Susan L. Zup, Creed M. Stary

**Affiliations:** ^1^ Department of Anesthesiology, Perioperative and Pain Medicine Stanford University Stanford California; ^2^ Developmental and Brain Sciences Program University of Massachusetts Boston Boston Massachusetts; ^3^ Department of Psychology University of Massachusetts Boston Boston Massachusetts

**Keywords:** extracellular matrix, hippocampus, interneurons, Parvalbumins, sex characteristics

## Abstract

**Introduction:**

Perineuronal nets (PNNs) are extracellular matrices that encompass parvalbumin‐expressing parvalbumin positive (PVALB+) fast‐spiking inhibitory interneurons where they protect and stabilize afferent synapses. Recent observations that gonadal hormones influence PVALB+ neuron development suggest that PNN regulation may be sexually dimorphic. Sex differences in PNN abundance and complexity have been reported in sexually dimorphic nuclei in zebra finch brains; however, corresponding differences in mammalian brains have not been investigated.

**Methods:**

In this study we assessed the number of cortical and hippocampal PNNs in juvenile and young adult male and female rats using fluorescent immunohistochemistry for PVALB and the PNN marker Wisteria Floribunda Lectin.

**Results:**

We report here that PNNs are numerous and well developed in hippocampal cornu ammonis‐1 of adult males but are lower in juvenile and possibly adult females. No significant differences were observed between sexes in cornu ammonis‐3 or adjacent neocortex. There was an observed developmental difference in the neocortex as juveniles had more PVALB+ cells, but fewer PNN+ cells, than adults.

**Conclusions:**

Because PNNs are integral for several hippocampal‐mediated learning and memory tasks, these observations have potential sex‐dependent translational implications for clinical strategies targeting cognitive dysfunction.

## 1 INTRODUCTION

Hippocampal perineuronal nets (PNNs) have been demonstrated to play a central role in neural plasticity, fear conditioning (Hylin, Orsi, Moore, & Dash, 2013; Thompson et al., 2018), and long‐term potentiation (Yamada & Jinno, 2013). PNNs regulate learning and memory *via* stabilization of inhibitory parvalbumin positive (PVALB+) interneurons (Yamada, Ohgomori, & Jinno, 2015), providing protection against physical forces and reactive oxygen species (Cabungcal et al., 2013). PNN expression peaks in adulthood but is minimal in newborn and aged animals (Yamada & Jinno, 2013). The appearance of PNNs in juveniles coincides with the closing of critical developmental periods (Cornez et al., 2018; Pizzorusso et al., 2002), helping to solidify active synaptic networks (Dityatev et al., 2007). Loss of PNNs in the brain has been linked to brain diseases associated with neurological and cognitive dysfunction, including epilepsy (McRae & Porter, 2012), schizophrenia (Shah & Lodge, 2013), and Alzheimer's disease (Morawski, Brückner, Jäger, Seeger, & Arendt, 2010).

PNNs have been shown to play a direct role in bird song learning and a general capacity for learning in juvenile males (Balmer, Carels, Frisch, & Nick, 2009). Concomitantly, several sexually dimorphic brain structures within the song learning and song production circuit also exhibit sex‐specific expression of PNNs, notably the HVC (cortex) and the robust nucleus of the arcopallium (amygdala homolog; Meyer, Boroda, & Nick, 2014). In mammals, PVALB hippocampal expression is regulated by, and positively correlated with, gonadal hormones, especially 17β‐estradiol (Wu, Du, Buuse, & Hill, 2014), which also provides protection to PVALB+ neurons (Rewal, Wen, Wilson, Simpkins, & Jung, 2005). In addition, exposure to gonadal hormones during perinatal development helps sculpt many sex differences in the mammalian brain (reviewed in Zup & Forger, 2017; McCarthy, 2016), so a sexual dimorphism may also exist in mammalian hippocampal PNN expression. Therefore, in this study we assessed cortical and hippocampal PNNs expression in male and female juvenile and young adult rats.

## 2 METHODS

### 2.1 Animals

All animal work was performed under the supervision of the University of Massachusetts Boston Institutional Animal Care and Use Committee. Male and female Sprague‐Dawley rats (postnatal day 18, *n* = 8 male, 6 female; postnatal day 52‐86, *n* = 7 male, 6 female) were sacrificed and brains fixed with 4% paraformaldehyde as previously described (Edwards, Madden, & Zup, 2018).

### 2.2 Immunohistochemistry and microscopy

Brains were sectioned in a cryostat at 35 μm and prepared for fluorescent immunohistochemistry as previously described (Xu, Ouyang, Xiong, Stary, & Giffard, 2015). Briefly, sections were treated with sheep anti‐parvalbumin (PVALB; [1:500]; R&D Signaling cat# AF5058) and the PNN‐binding fluorescent wisteria floribunda agglutinin (WFA; [1:500]; Vector Labs cat# FL‐1351) and incubated in 594 nm secondary (Invitrogen cat# A‐11016). Z‐Stacks (1μm thick X 35 sections) were acquired through cornu ammonis‐1 (CA1), cornu ammonis‐3 (CA3), and somatosensory cortex using a Zeiss Imager M2 equipped with an Apotome 2 for optical sectioning and collapsed into a maximum‐projection image for cell quantification. Average PVALB+ and PNN+ cell counts were acquired from six hippocampal sections per animal, using maximum projection images by an individual blinded to experimental group. Neocortical cell counts were acquired from the same sections in the adjacent somatosensory/barrel cortex, dorsolateral to the rostral hippocampus, in order to limit variability in PVALB+ and PNN+ counts that could be due to tissue processing.

### 2.3 Statistics

Cell counts were analyzed with a two‐tailed independent samples *t* test using SPSS (IBM version 22). A *p* value <0.05 was considered statistically significant.

## 3 RESULTS

### 3.1 Hippocampus

**Figure 1:**
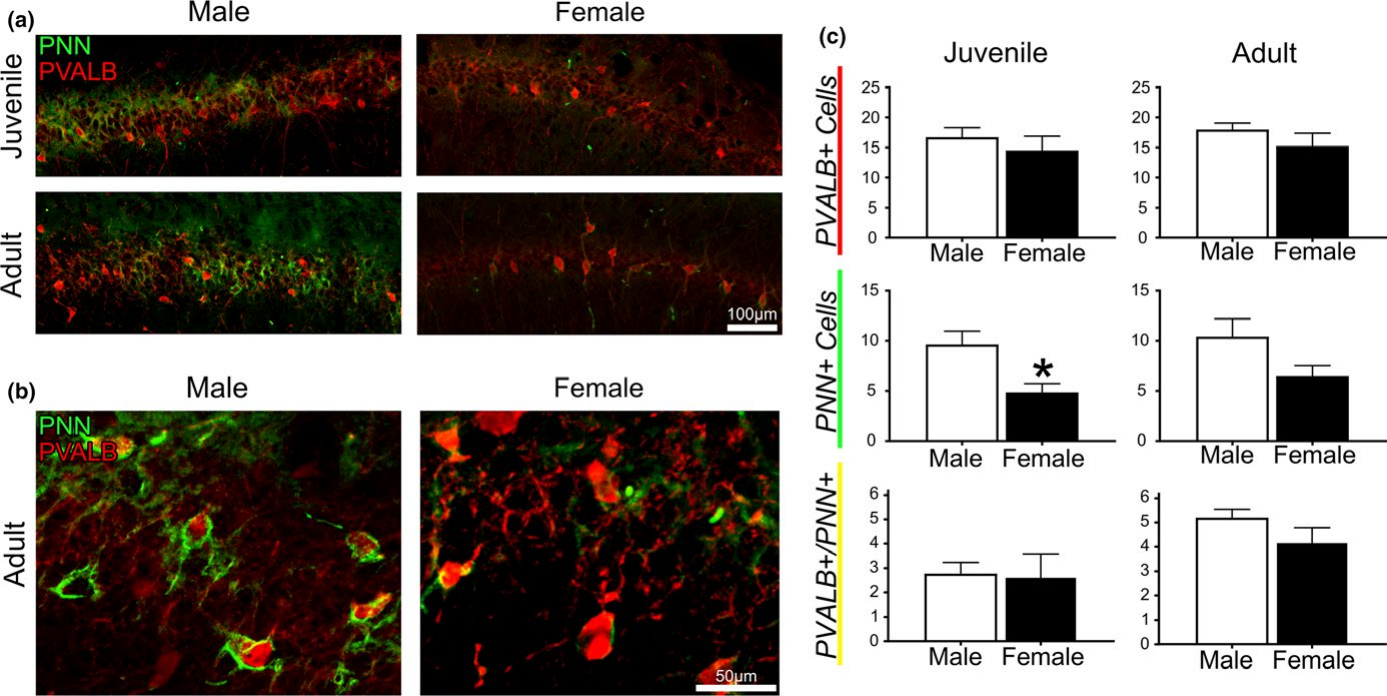
Hippocampus. (a) Representative images from cornu ammonis‐1 (CA1) of each group. (b) Enlarged views of representative images of adult male versus adult female perineuronal nets (PNNs). (c) Mean count of hippocampal parvalbumin positive (PVALB+) interneurons and neurons with PNN+ in juvenile (left) and adult (right) male and female rats. *p < 0.05, Bars represent mean ± SEM

Our analysis assessed the total hippocampus, as well as CA1 and CA3 sub‐regions, in males and females at two different ages. There were no significant differences in any measures for total hippocampus or for CA3. For CA1, there were no significant differences in the average PVALB+ interneuron count between age (*p* = 0.66) or sex (*p* = 0.17; Figure 1). However, male juvenile rats exhibited nearly twice as many PNNs in CA1 than female juvenile rats (9.63 ± 3.80 vs. 4.86 ± 2.14; *p* = 0.02; Figure 1c). In adult animals, CA1 PNN counts trended in a similar fashion (10.42 ± 4.68 in males vs. 6.45 ± 2.58 in females) however the difference was not statistically significant (*p* = 0.09). There were no differences between adults and juveniles when separated by sex in either males (*p *= 0.72) or females (*p *= 0.11). Figure 1 represents hippocampal CA1 PVALB+ count, PNN count, and PVALB+/PNN+ co‐labeled cells in all groups. Qualitatively, WFA staining was more intense and well defined in adult male CA1 versus female CA1, where fluorescence intensity was faint and PNNs did not always fully envelop the PVALB+ cells (Figure 1b). WFA staining in juvenile animals was similar between the sexes.

### 3.2 Neocortex

**Figure 2:**
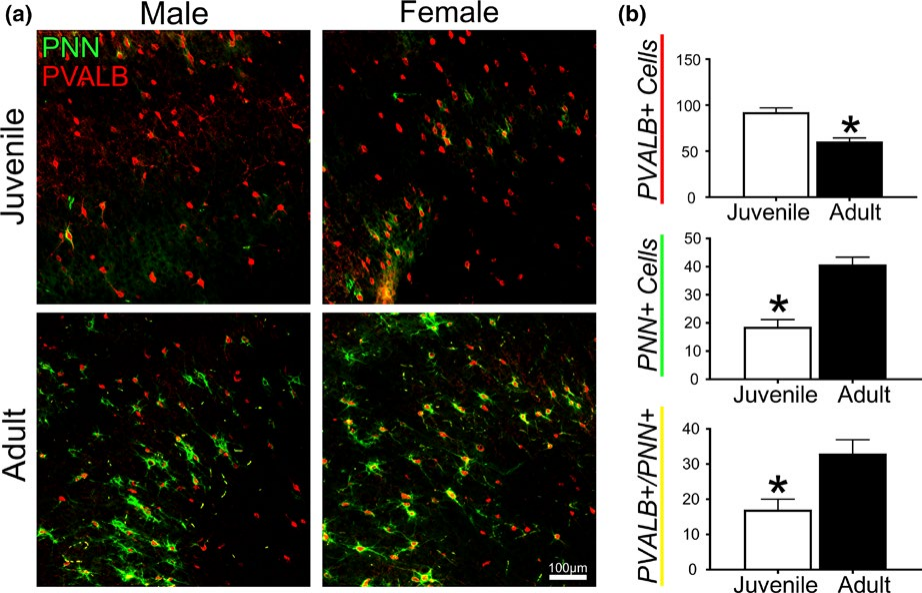
Neocortex. (a) Representative images from each group. (b) Mean count of cortical parvalbumin positive (PVALB+) interneurons and neurons with perineuronal nets (PNN+) in juvenile and adult rats. *p < 0.05, Bars represent mean ± SEM

Figure 2 outlines observations in PNN and PVALB expression in the neocortex where PNNs are commonly studied (Wang & Fawcett, 2012). There was no overall sex difference in PVALB+ cell count between males (81.00 ± 19.25) and females (71.88 ± 20.10) or in PNNs between males (32.50 ± 12.90) and females (26.88 ± 13.89). However, we observed significant (*p *= 0.002) differences in both PVALB+ and PNN+ cell number between developmental time points. The neocortex of juveniles had nearly twice the number of parvalbumin neurons (92.38 ± 9.52) as adults (60.50 ± 7.88). Despite the large amount of PVALB+ interneurons, juvenile rats had less than half the number of PNN+ cells observed in adults (18.63 ± 5.19 versus 40.75 ± 5.24; *p *< 0.001). In contrast to observations in hippocampus, both male and female adult rats displayed robust cortical PNN staining and fully enveloped PVALB+ cells (Figure 2b).

In both the hippocampus and neocortex, the percentage of PVALB+/PNN+ co‐labeling did not differ between adult and juvenile females (*p *= 0.31). However, juvenile male rats exhibited a lower percentage of co‐labeling than adult males in the hippocampus (10.48 ± 3.09 vs. 6.84 ± 2.49, respectively) as well as in neocortex (38 ± 3.54; vs. 20.75 ± 1.06, respectively).

## 4 DISCUSSION

This study is the first to report sex differences in mammalian hippocampal and cortical PNN expression. In addition to the differences between sexes, we also report that male rats show greater differences in PNN expression and PVALB co‐expression between developmental time points than do female rats. Several recent studies on PNNs have included both sexes in their experiments but did not conduct analyses between sex or comment on the presence or absence of sex differences (Chu et al., 2018; Saito et al., 2018; Yukawa et al., 2018). We report a male‐biased sex difference in CA1 PNN expression by postnatal day 18, along with a trend for a similar pattern in adulthood. These findings are specific to CA1 in this restricted analysis, as no sex differences were observed in CA3 or the adjacent neocortex.

The sex‐specific expression of PNNs in CA1 may relate to observations of sex‐specific hippocampal function. For example, male hippocampal neurons have been shown to be more susceptible to oxidative injury (McCullough, Zeng, Blizzard, Debchoudhury, & Hurn, 2005), which may necessitate their increased PNN protection. More generally, developmental maturation is often sexually dimorphic, and our observations suggest earlier CA1 PNN envelopment of PVALB+ interneurons in males at PND18. PNNs contribute to the maturation of excitatory networks by modulating inhibition (Dityatev et al., 2007), and our observations may hold relevance to prior observations that juvenile males are more vulnerable to overexcitation and excitotoxicity during development (reviewed in McCarthy, 2016).

In addition to the juvenile sex differences in quantitative PNN expression, we observed that adult males display robust hippocampal PNN labeling, while PNN expression in adult females and juveniles of both sexes was more variable. Fluorescence intensity of WFA staining has been proposed as a proxy of PNN maturity and function (Koppë, Brückner, Brauner, Härtig, & Bigl, 2016), suggesting these observations may indicate a difference in PNN regulation and function, implying differences in the underlying mechanisms regulating learning and memory between males and females. The observations in this study may hold clinical relevance as PVALB+ neurons have been shown to modulate drug responses in a sex‐ and anxiety trait‐specific manner (Ravenelle, Neugebauer, Niedzielk, & Donaldson, 2014), and PNNs are thought to modulate the response to common antidepressants (Karpova et al., 2011).

One limitation of this study was the limited sample size, and it is likely that the abundance of PNNs in the CA1 of adult females would also be significantly lower than adult males with a larger sample size. Both parvalbumin and PNN development are highly region‐specific (Ueno et al., 2018); however, in most cases PVALB+ neurons increase with age. Our finding that juvenile rats had nearly twice the number of PVALB+ interneurons in the neocortex compared to adults is in sharp contrast to many previous studies, however it is consistent with a prior report in the barrel cortex of mice (Nowicka, Soulsby, Skangiel‐Kramska, & Glazewski, 2009). This further suggests that PNN and PVALB expression are highly region‐specific. The observations in this study suggest that a more comprehensive, whole brain assessment may be warranted to definitely assess the spatial role PNNs play in neurodevelopment. Given the far‐reaching effects of PNNs on neurophysiology and their emerging prominence in neurological disorders (Wen, Binder, Ethell, & Razak, 2018), it is possible that even small differences in PNN abundance, especially during development, contribute to sex‐dependent effects in the hippocampus. As interest in PNNs is quickly rising, current and future studies investigating the role of PNNs in mammalian neuroplasticity should include consideration of age and sex.

## CONFLICT OF INTEREST

None.
